# Meta-analysis of public health risks of lead accumulation in wastewater, irrigated soil, and crops nexus

**DOI:** 10.3389/fpubh.2022.977721

**Published:** 2022-10-18

**Authors:** Adane Sirage Ali, Argaw Ambelu Bayih, Sirak Robele Gari

**Affiliations:** ^1^Department of Urban Environmental Management, Kotebe University of Education, Addis Ababa, Ethiopia; ^2^Department of Water and Public Health, Institute of Ethiopian Water Resources, Addis Ababa University, Addis Ababa, Ethiopia

**Keywords:** lead (Pb), wastewater, health risk index, lead accumulation, irrigated soil, meta-analysis, wastewater irrigation, heavy metal accumulation

## Abstract

Lead (Pb) from different sources accumulate in the irrigation water, irrigated soil and in different parts of plants. Reports show contradictory findings and high variability of lead accumulation and associated public health risks. We hypothesized that lead accumulation in irrigation water, soil and edible plants is high enough to be a public health risk. By using the standard procedures for meta-analysis, 24 studies were qualified. The studies included in the meta-analysis are concentrated in few countries with strong authors' key words co-occurrence relationship. The mean concentration of Pb in the irrigation wastewater ranged from 0.0196 ± 0.01 mg/l to 52.4 ± 0.02 mg/l in wastewater and about 50% of the values are beyond the limits for irrigation water standard. The study also showed that the concentration of Pb in the irrigated soil vary significantly from a minimum of 0.04 ± 2.3 mg/l in Ethiopia to a maximum of 441 ± 19.8 mg/l in Iran (*P* < 0.01). Based on effect size analysis, the weight of the studies ranged from 0.1 to 5.4% indicating that the studies' contribution to the overall effect is barely different. The heterogeneity test statistics also indicates considerable variability between the studies (I^2^ = 98%, *P*-value < 0.001). The subgroup analysis showed large between-studies heterogeneity in both groups (Tau^2^ = 28.64; T^2^ = 98%). A total of 44 crops were studied, of which 38 were leafy and non-leafy vegetables. Most popular crops including spinach, cabbage and lettuce are most frequently studied crops. In all crops, the Pb level in crops produced by using untreated wastewater are beyond the WHO limit for edibility. In all of the studies, the pollution load index (PLI) and soil accumulation factor (SAF) is much higher indicating that there is a buildup of Pb concentration in wastewater irrigated soil. The plant concentration factor (PCF) calculated shows the high Pb accumulation potential of the edible parts of the crops. The health risk index (HRI) calculated shows that in all of the studied crops from India, Iraq, Morocco and Egypt are much higher than one indicating the high health risk of consumption.

## Introduction

Recently a number of articles have been published on wastewater-irrigated soils and crops contamination with heavy metals (1–9). Lead [Pb (II)] found in wastewaters comes from many sources such as metal plating, tanneries, oil refining, and mining. Once lead reaches the environment, it spreads through soil and water streams and accumulates in the body through the food chain, resulting in a high risk to human health (10). It has been reported that severe exposure to lead is associated with sterility, abortion, stillbirths, and neonatal deaths (11). Exposure to toxic heavy metals including lead due to prolonged consumption of contaminated foods or occupational ingestion or inhalation of irrigated soil, wastewater aerosols, and irrigated produce is linked to a wide range of chronic health effects (12, 13). Consumption of vegetables, grains, fruits, and animal meat containing heavy metals in the short or long term can endanger the health of consumers, causing public warnings and epidemiology concerns (14, 15).

Several studies indicated that cereals and vegetables cultivated in soils contaminated by different pollutants contain a significant amount of lead together with other toxic heavy metals (16–18). Pb bioaccumulation in plants is mainly attributable to the pollution of irrigation water and soil (19), particularly the use of wastewater for food crop production. Several studies show variabilities among various crops in their capacity to accumulate various types of heavy metals (20–24). Few studies reported low levels of lead in vegetables produced using wastewater, though the concentration in the wastewater and irrigated soils is high. Others reported even higher levels of lead in vegetables than in wastewater itself and wastewater-irrigated soils.

Despite several studies have been carried out to assess the concentration of heavy metals in wastewater, irrigated-soil, and edible parts of various crops, there haven't been systematic reviews and meta-analyses which emphasizes lead accumulation in wastewater irrigation systems. Studies focusing only on lead bioaccumulation and associated risk levels is almost negligible. Reported risk levels of lead bioaccumulation in wastewater-based vegetables vary by plant types, geography, and analysis. Publications released different views about the public health risks of using wastewater-produced crops. Thus, there is a need to synthesize different finding reports and views on potentially damaging aspects of Pb.

Therefore, the main objective of this study is to carry out a meta-analysis on the accumulation of lead in wastewater, wastewater-irrigated soils and wastewater-based vegetables; and associated public risks.

## Methods

### Review questions and protocols

The review attempts to answer the following core review questions: How does lead concentration vary in wastewater, wastewater-irrigated soil, and crops in different countries? Does the re-use of wastewater for irrigation significantly contribute to the natural lead concentration in irrigated soil and then lead to increased bioaccumulation in crops? Which crops are most commonly grown in wastewater irrigation and preferred for heavy metal risk assessment? Does lead concentration in the wastewater-based crops is beyond the WHO/FAO standards for edible vegetables. Do all examined edible vegetables accumulate lead high enough to become a public health risk?

### Search strategy

Studies of potential interest were identified by creating a comprehensive search algorithm. The first step was defining key terms by reviewing the final verified title and this was in turn, used to choose appropriate databases. Terms were then combined in relevant categories using Boolean logic operators (“OR” and “AND”). The search procedure was tested by the chosen databases: PubMed (https://www.ncbi.nlm.nih.gov/pmc/) and Science direct (https://www.sciencedirect.com); and then the final algorithm that retrieved the highest proportion of all known relevant articles was selected: *Heavy metals* or *Lead in wastewater irrigation, public health risks of wastewater irrigation*. Gray literature was identified using internet-wide search engines [Google (https://www.google.com/)] and Google Scholar (https://scholar.google.com/).

### Citation selection—inclusion and exclusion of articles

First, citations identified via the search engine were screened by title relevance and imported into the reference management program (EndNote 20). The next step-by-step citation selection processes until the final qualification for analysis were done by using the reference manager platform. By using Endnote, duplicates were removed by the automatic de-duplication option. Then, studies were filtered by using the databases' tool such as “research articles published in English language, published between the years 2000 and 2021, and original articles”. The remaining outputs were screened at individual article title level: by the presence of abstracts and full text, and by the prior set inclusion and exclusion criteria. The inclusion criteria include “*each article must report Pb concentration (mean* ± *standard deviation) in the irrigation wastewater, irrigated soil/dosed amount, and edible parts of vegetables in both experimental and control groups. Sample size of each measurements and for how long the land is irrigated should also be reported*.”

**Figure 1:**
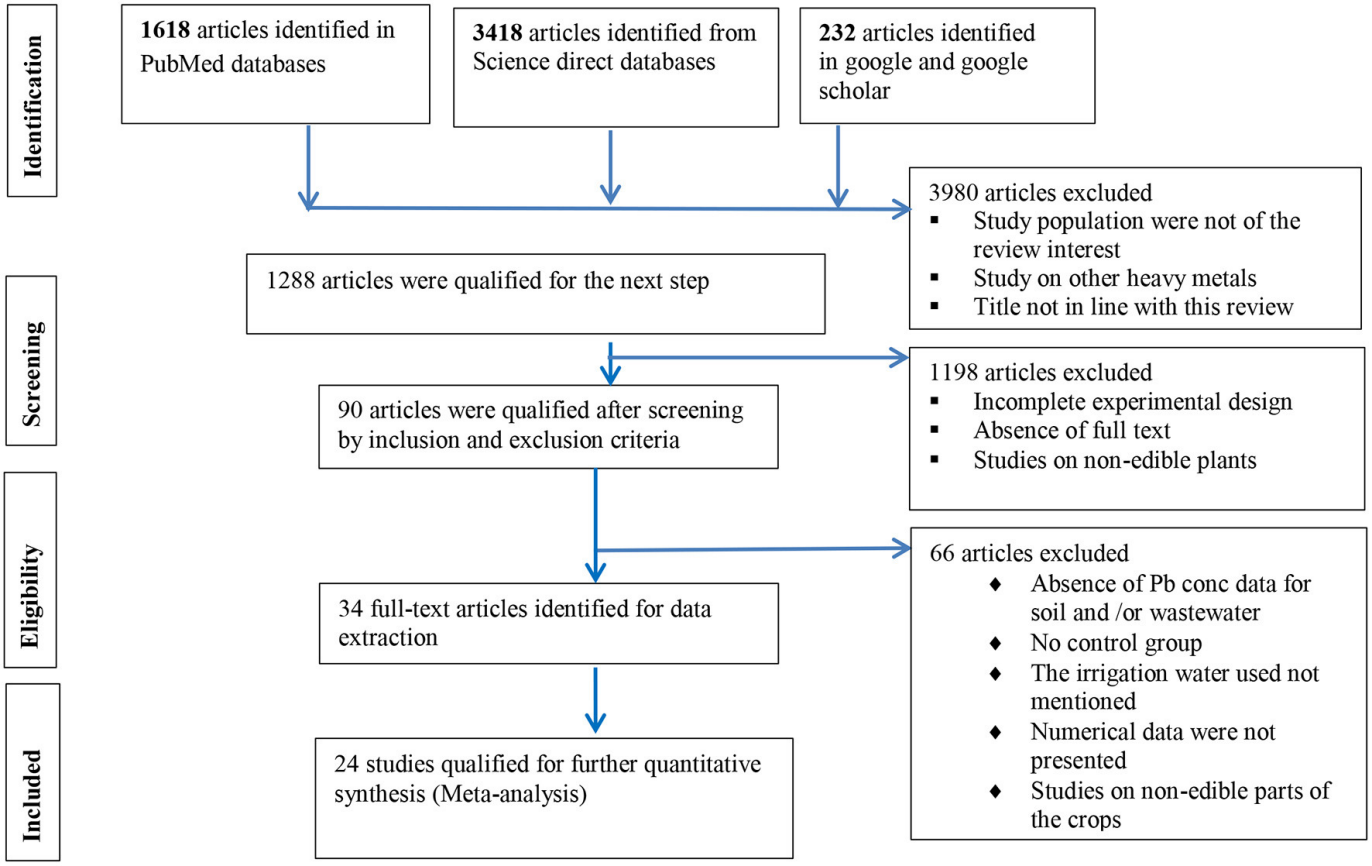
A PRISMA flowchart outlining the study selection process.

The number of articles disqualified for this review together with the reasons for rejection was carefully recorded, and finally, the whole study selection process was generated in diagram by Prisma 2009 generator using the online platform (Figure 1).

### The search output

The overall search strategy and article selection process are presented by using Prisma 2009 diagram (Figure 1). The two databases (PubMed and science direct) and google scholar produced 5268 articles. After screening by title relevance and removing the duplicates, the search strategies (electronic databases) produced 3288 articles. Then, the outputs were screened by using the following exclusion criteria and only 90 articles were qualified for data extraction.

- Did the articles include lead (Pb) while assessing other heavy metals?
- Is the article original research?
- Is the title in line with the review interest?

From the remaining 90 studies that qualified for full-text screening, during data extraction, 66 of the articles were rejected because of the following reasons.

- The absence of a control group in the experimental design.
- Absence of lead concentration data for the irrigated/control soil or wastewater.
- The irrigation water type used was not clearly mentioned.
- Numerical data were not presented, instead, the data were presented graphically and the efforts made to extract from the graph or to get the data from the authors were not successful.

Geographically, the studies were mainly concentrated in Asian countries particularly countries with large population sizes such as India, Iran, China, and Pakistan, which are the largest untreated wastewater users in the world (25). Although Latin American nations such as Chile and Peru are among the largest wastewater users in the world, studies are not included in this meta-analysis because of the language they published is not English.

### Data collection and analysis

#### Data extraction

A piloted data extraction tool was used to extract data from each study that met the inclusion criteria. From each article, information were extracted based on: the authors, publication year and country; the sample size and the mean concentrations with a standard deviation of Pb in the wastewater, control irrigation water, irrigated soil and edible parts of the crops; the type of wastewater used and how long the farmland irrigated; the lists of crops studied; and the instrument used for Pb quantification.

#### Data analysis

Meta-analysis was carried out by using Stata software, version 16.0 (Stata Corp, College Station, TX, USA), Cochrane RevMan 5, MedCalc version 20, and Microsoft excel sheet. Effect sizes were generated by the random-effect model because the studies included in this meta-analysis were not expected to estimate a common effect size due to the differences in study locations, conditions, experimental setups, and methods used in the individual studies (26). For each uncontaminated-to-contaminated comparison of the irrigation water and irrigated soils, the unbiased standardized mean difference (Hedges' *g*) as a common effect size was calculated. The mean effect size was considered statistically significant if the 95% bootstrap confidence interval did not include zero.

The effect of the duration of irrigation on the Pb concentration of the irrigated soil was estimated by using meta-regression by taking years of irrigation as a moderator. To estimate the heterogeneity among different studies, I^2^ and Tau^2^ statistics were applied.

#### Health risk assessment

The potential health risk of Pb consumption through vegetables were assessed based on the bio-concentration factor (BCF), daily intake of metal (DIM) (27), and HRI (28). Moreover, lead PLI and soil lead accumulation factor (SAF) were also calculated.

• **Health risk index:** The HRI through the consumption of contaminated vegetables were assessed based on the food chain and the reference oral dose (RfD) for each metal. The HRI < 1 means the exposed population is assumed to be safe.
  HRI = DIM/RfD = daily intake of metals/reference oral dose (29). RfD of Pb for adults is 0.001.
• **Daily intake of lead:** Daily intake of Pb is calculated based on the Pb concertation in the food and weight of the person.

DIM = (C_metal_ × C_factor_ × D_foodintake_)/B_averageweight_, Where C_metal_, C_factor_, D_food_ _intake_, and B_averageweight_ represent the Pb concentrations in plants (mg/kg), conversion factor, daily intake of vegetables, and average body weight, respectively. The conversion factor 0.085 was used to convert fresh green vegetable weight to dry weight (30). The average daily vegetable intakes for adults is considered to be 0.345 person/day, when the average adult body weights is considered to be 55.9 kg, as used in previous studies (31).

• **The bioaccumulation / bioconcentration factor:** BCF = C_plant_/C_soil_, (32), where C_plant_ and C_soil_ represent the heavy metal concentration in extracts of plants and soils on dry weight basis, respectively.
• **The pollution load index (PLI):** The degree of soil pollution for each metal was measured using the PLItechnique depending on soil metal concentrations. The following modified equation was used to assess the PLI level in soils.

PLI = C (sample soil)/C (reference soil), (33).

- **Soil accumulation factor (SAF):** The SAF was also calculated by using the following formula.

SAF = C_soil_/C_wastewater_

where C_soil_ and R_eference_ represent the heavy metal concentrations in the wastewater-irrigated soil and wastewater, respectively.

## Results and discussion

### Bibliometric analysis of the key words

**Figure 2:**
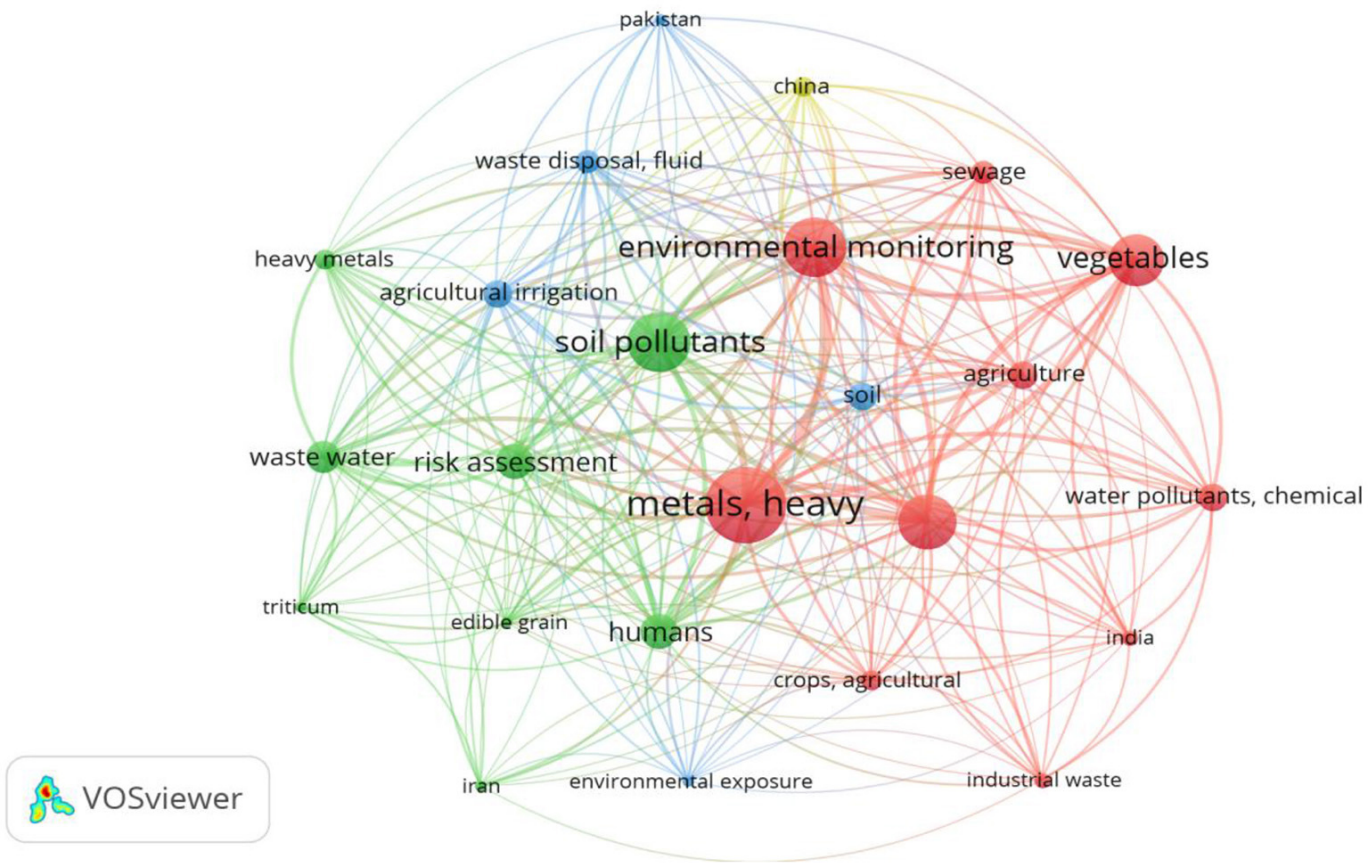
Bibliometric analysis of the co-occurrence of keywords in the selected publications.

Figure 2 shows the bibliometric analysis of the co-occurrence of keywords in the publications included in this meta-analysis. Keywords provided by authors of the paper and occurred for more than 3 times in the VOSviewer core database were enrolled in the final analysis. Of the 94 keywords used by the authors, 71 met the threshold. The shorter the distance between two nodes, the larger the number of co-occurrence of the two keywords (34). The first three keywords that appeared most as a keyword were “metals, heavy” with a total link strength of 125, “environmental monitoring” with a total link strength of 103, and “soil pollutants” with total link strength of 98. These key words were mostly used in articles published between 2012 and 2014. Four nations, where most of the articles are selected, are also used as key words: China, India, Pakistan and Iran. The size of nodes associated with the keywords in the diagram indicates the frequency of occurrence. The curves between the nodes represent their co-occurrence in the same publication.

**Figure 3:**
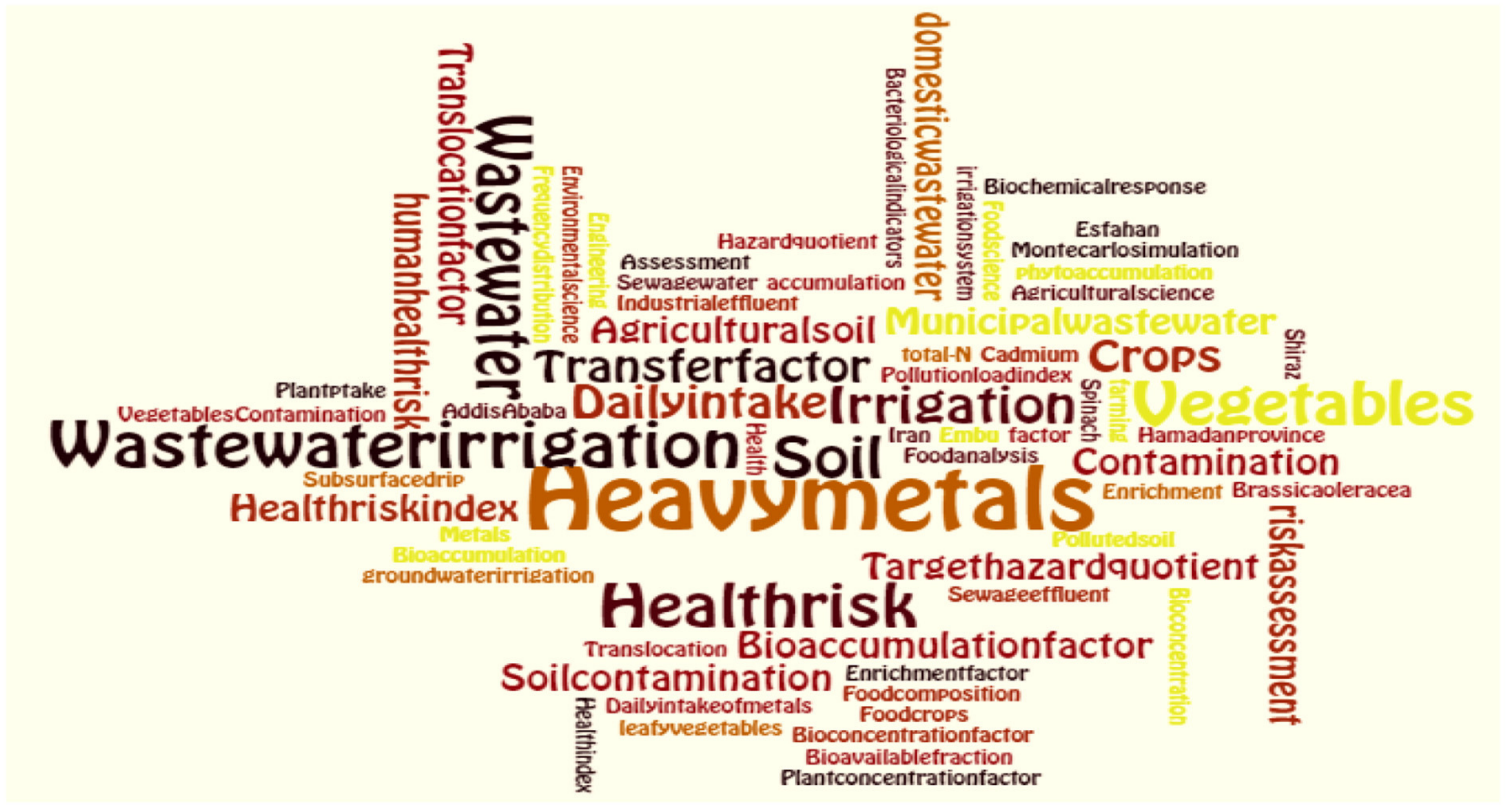
Word cloud of 91 keywords used by the authors in the selected studies.

A word cloud is also created to show the frequency of the 91 keywords of the authors which occurred for more than 10 times. The font size of the key words in the word cloud represent the frequency of occurrence (35). It was indicated that “heavy metals” was the most frequent word used as a key word followed by “wastewater irrigation”, “irrigation,” “vegetables,” and “health risk” (Figure 3).

### Distribution and major characteristics of the studies

The Twenty Four studies qualified for the meta-analysis are conducted in thirteen countries. Among these, Seventeen of them were obtained from four countries: India, China, Iran and Pakistan. International Water Management Institute (36), by citing different media outlets and new research findings, reported that from 35.9 Mha of land irrigated by untreated wastewater, majority of the irrigated land is accounted for India, China, Pakistan, Mexico and Iran. In line with these reports, this study also showed that about 71% of the studies are carried out in four countries: India, Pakistan, Iran and China (Table 1).

**Table 1:** The number of studies by geographical location and years of publication.

**Countries**	**Number of studies**	**Publication year**	**Number of articles**
India	6	2006	1
Pakistan	5	2007	1
Iran	4	2008	2
China	2	2011	3
Botswana	1	2012	1
Ethiopia	1	2013	1
Egypt	1	2014	3
Kenya	1	2015	3
Iraq	1	2017	2
Morocco	1	2018	1
Nigeria	1	2019	2
N. Korea	1	2020	3
S. Korea	1	2021	2

Table 2 shows the major characteristics of the studies included in the meta-analysis. The studies selected for the meta-analysis were carried out by a total of 87 authors from 13 countries (2 continents). In eighteen of the researches, untreated municipal wastewater was used whereas in six of them, treated wastewater was used. For the measurement of Pb concentration in the irrigation water, irrigated soil and vegetables, atomic absorption spectroscopy (AAS) was used in nineteen of the studies whilst in three studies, FAS, and in two studies, GF-AAS were used. AAS is the most frequently used tools in the studies of analytical chemistry. This is because for the determination of most metals and metalloids the technique offers sufficient sensitivity for many applications and is relatively interference free (56). In the study, 47 crops (40 vegetables and 7 seed plants) were included. From the 24 studies, 17 of them were studied in four nations namely: India (6), Pakistan (5), Iran (4) and China (2). In a single study from one to a maximum of 12 crops were studied at a time (23) (Table 2).

**Table 2:** Major characteristics of the studies (Author and year of the studies).

**Author/authors**	**Country**	**Wastewater type**	**Methods of detection**	**Crops studied**
Asgari and Cornelis (3)	Iran	Treated	AAS	Corn and wheat
Chung et al. (37)	N. Korea	Untreated	AAS	Broad beans, Durum Wheat, soft wheat, Oat, Nettle, Broadleaf, plantain, Alfalfa and Mallow
Gupta et al. (38)	India	Treated	AAS	lettuce, pudina, celery, cauliflower, spinach, coriander, parsley, Chinese onion and Radish
Khan et al. (23)	Pakistan	Untreated	FAAS	Coriander, Onion, Okra, Garlic, Capsicum, Carrot, Brinjal, Spinach, Radish, Mint, Tomato and Wheat
Khan et al. (4)	China	Untreated	GF-AAS	Radish„ maize, green cabbage, spinach, cauliflower, turnip, and lettuce
Kharazi et al. (39)	Iran	Untreated	AAS	Persian leek, basil, lettuce, potato, and tomato
Kim et al. (40)	S. Korea	Treated	AAS	lettuce, squash, cucumber, tomato, Chinese cabbage, and radish
Letshwenyo and Mokokwe (41)	Botswana	Treated	AAS	Spinach
Mahfooz et al. (42)	Pakistan	Untreated	AAS	Corn, rice, sugarcane, millet, wheat
Mustapha and Adeboye (43)	Nigeria	Untreated	AAS	Spinach
Qishlaqi et al. (44)	Iran	Untreated	AAS	Wheat, lettuce, spinach, celery
Rezapour et al. (45)	Iran	Treated	AAS	Wheat
Sayo et al. (46)	Kenya	Treated	AAS	Spinach
Tariq (47)	Iraq	Untreated	AAS	Chard, leek, celery and cress
Tiwari et al. (6)	India	Untreated	F-AAS	Spinach, Radish, Tomato, Chili, Cabbage, Okra, coriander, drill, cabbage, Cress
Ullah and Khan (48)	Pakistan	Untreated	AAS	Cabbage
Ullah et al. (49)	Pakistan	Untreated	AAS	Spinach
Wang et al. (50)	China	Untreated	AAS	Chinese cabbage, cabbage, lettuce, rape, scallion, radish, cauliflower, Leek
Woldetsadik et al. (51)	Ethiopia	Untreated	GF-AAS	Kale, Ethiopian Cabbage, Lettuce, chard
Ahmed and Slima (52)	Egypt	Untreated	AAS	Jew mallow
Chopra and Pathak (53)	India	Untreated	AAS	Beet, spinach, cauliflower, French beans
Sharma et al. (54)	India	Untreated	F-AAS	Amaranthus, cabbage, pallak, okra, tomato, egg plant
Waheed et al. (55)	Pakistan	Untreated	AAS	Spinach, lettuce

### Concentration of Pb in wastewater

**Figure 4:**
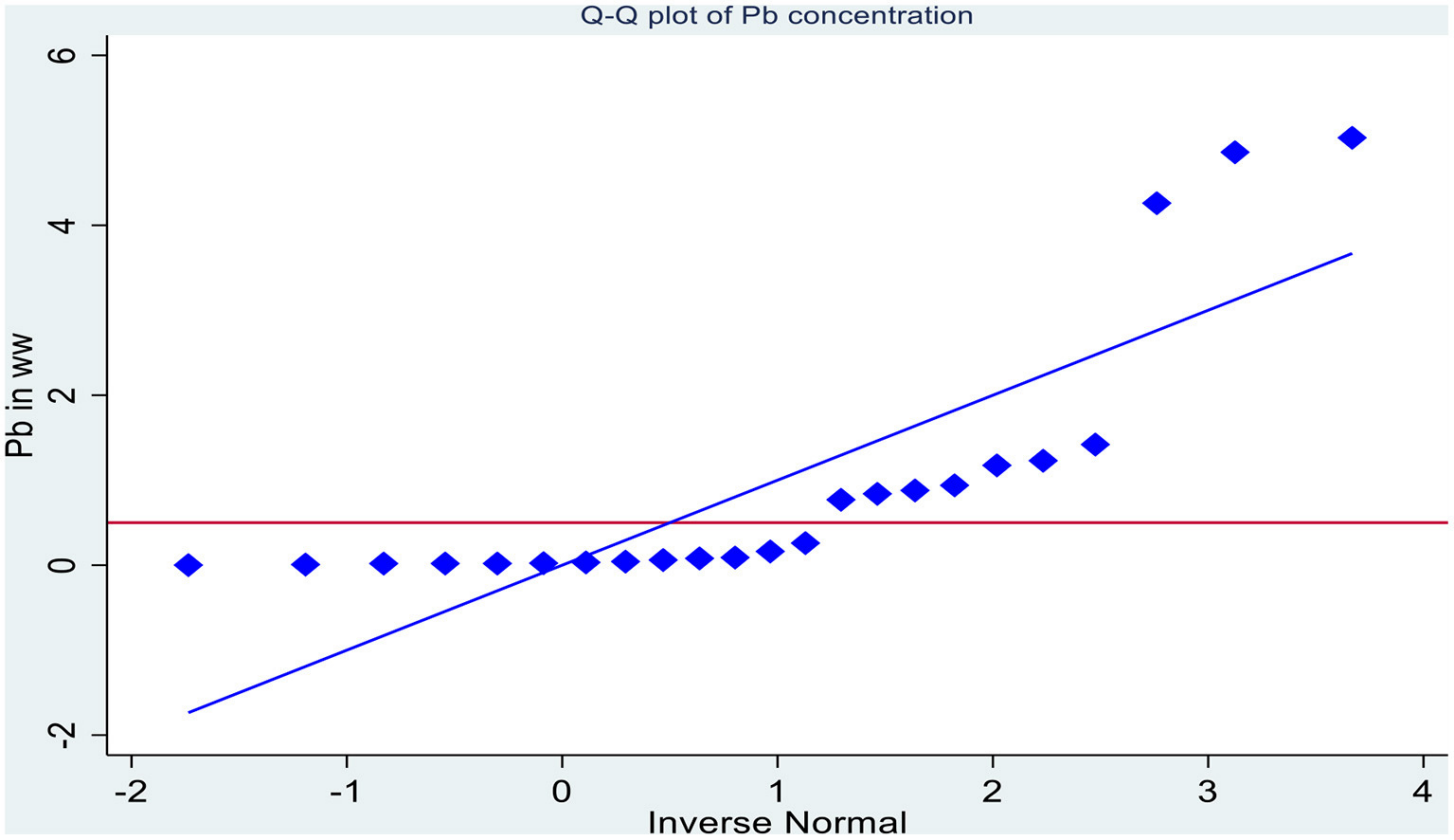
The data distribution of Pb concentration in irrigation wastewater compared with the WHO/FAO limits of Pb level in irrigation water (0.5).

There are several natural and man-made factors that can affect the concentration of lead in municipal wastewater. As a result, the amount of lead in wastewater varies from country to country, from region to region, and from time to time. The quantile plot shows the actual value of individual observation in the group making it easy to spot some unique values/higher values of Pb concentration (6, 23, 38) (Figure 4). The plot shows that the concentration of Pb in the irrigation wastewater is under-dispersed (platykurtic distribution) compared to normal distribution with few extreme outliers on both sides. In this study, the mean concentration of Pb in the irrigation wastewater ranged from 0.0196 ± 0.01 to 52.4 ± 0.02 mg/l in raw wastewater and 0.001 mg/l to 0.88 ± 0.13 mg/l in treated irrigation wastewater. The maximum concentration of Pb in the irrigation wastewater was recorded in Egypt in an experimental plot, which is not indicated in the Q-Q plot (52). The plot shows three distinctive data distribution: below 0.5, close to 0.5 (0.84–1.175) and much higher than 0.5 (4.26–52.4) mg/l.

The Pb concentration in the irrigation wastewater was compared with the WHO/FAO standards for Pb concentration in irrigation water (0.5 mg/l) using one-sample *t*-test and the analysis showed that there is significant difference between the Pb concentration in the wastewater and the limit set by the WHO/WHO (*P* < 0.05). Household items such as tap water, soap, toothpaste, detergents, and metal utensils, corrosion of metal, metal pipelines, metal containers, medicines, laundry, paints, batteries, cosmetics, garden products, glass, and food (grains, nuts, veggie, and tea leaves) can be the sources of hazardous elements in domestic wastewater (47, 57).

### Lead concentration in irrigated soils

Although Pb occurs naturally in the environment, a number of human activities can contribute; and thus affect the natural Pb concentration in the environment. In irrigated soil, a number of factors including the type of irrigation water can affect Pb concentration. Municipal wastewater may contain Pb from different sources and thus, soil irrigated by this kind of water can possibly contain increased levels of Pb. Our study shows that concentration of Pb in the studies vary considerably from a minimum of 0.04 ± 2.3 mg/l in Ethiopia (51) to a maximum of 441 ± 19.8 mg/l in Iran (44).

**Figure 5:**
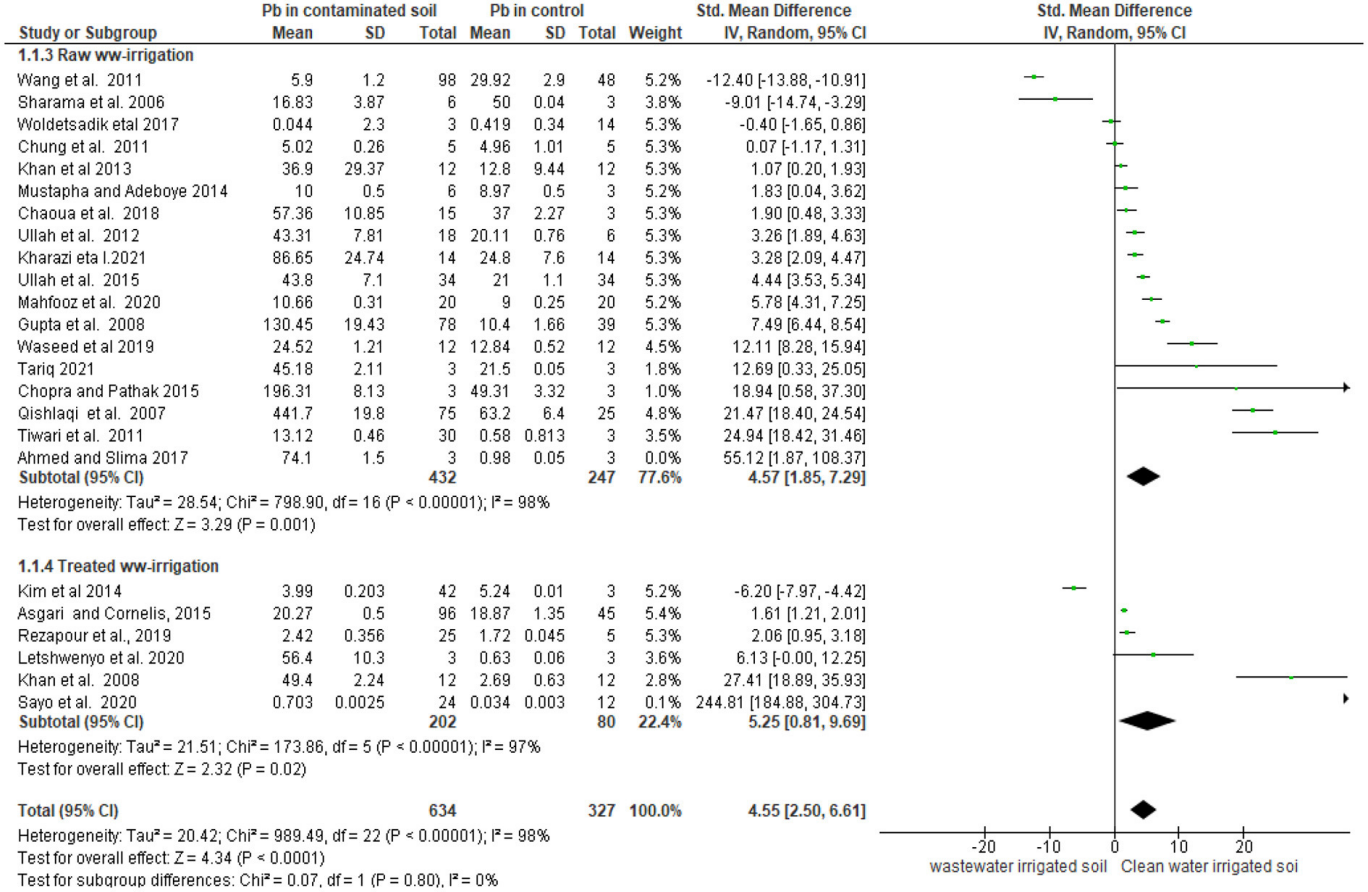
Mean effect sizes of random-effect models (mean Hedges' *g* ± 95% confidence interval) for Pb accumulation in wastewater irrigated soil vs uncontaminated soil. Values in brackets refer to the number of comparisons from which the mean effect size was calculated. A negative *g* value means higher Pb concentration in wastewater irrigated soil than uncontaminated sites. The mean effect size was considered statistically significant if the 95% bootstrap confidence interval (CI) did not include zero. CIs of continuous measures that include 0 represent no significant results.

Figure 5 presents the forest plot incorporating the effect size of each studies under two subgroups, confidence interval with *P*-values and study weights incorporated in a forest plot. The wider horizontal line in the forest plot (wide confidence interval) in five of the studies (4, 6, 41, 52, 53, 58) shows the smaller sample size and the non-significant differences between the treatment and the control groups implying that the mean Pb concentration of contaminated and non-contaminated irrigation water is not statistically significant. Based on the analysis, the weight of the studies ranged from 0.1 to 5.4% where majority of the studies (58.3%) have a weight between 5.2 and 5.4% indicating that the studies contribution to the overall effect of the study barely differed. In three of the studies (37, 41, 59), the horizontal line crosses the line of the null effect (Figure 5) indicating that the line of null effect value lies within the confidence interval and thus it could be the true value; therefore, these studies do not illustrate a statistically significant difference between the Pb concentration in wastewater irrigated soil and non-wastewater irrigated soil. Three studies (40, 50, 54) showed statistically significant negative effect, but the rest of the studies (eighteen of the studies) showed statistically significant positive effect.

#### Between-study heterogeneity

The between-study variation of the effect sizes is evident from the forest plot. The heterogeneity test statistics indicates considerable variability between the studies (I^2^ = 98%, *P*-value < 0.001), which indicates that the studies are “considerably heterogeneous” (60). When the Q value result is significant (*p* < 0.05), it is an indication that the studies are heterogeneous. For heterogeneity values >50%, the random effect model is appropriate (61). This very high proportion of observed variance that reflects real differences in effect size suggests that the studies in this meta-analysis cannot be considered to be studies on the same population and thus it is worthwhile to further discuss subgroup and moderator analysis. Moreover, Tau, a measure of the dispersion of the true effect sizes between studies in terms of the scale of the effect size (62), also showed large heterogeneity between the studies (Tau^2^ = 20.48) indicating that the wide distribution of the standard deviations of the true effect sizes under the assumption that these true effect sizes are normally distributed.

#### Subgroup analysis

The subgroup analysis was carried out by categorizing the studies based on the type of wastewater used for irrigation. Seventeen studies used raw wastewater whilst the other six used treated wastewater for irrigation. The analysis showed large between-studies heterogeneity in both groups (Tau^2^ = 28.64; T^2^ = 98% and Tau^2^ = 21.51; T^2^ = 97% for studies based on raw wastewater-irrigated soil and treated wastewater soil, respectively) (Figure 5). The subtotal effect sizes of each subgroup show that there is significant between-study differences in both raw wastewater and treated wastewater groups. However, the between-studies difference among treated wastewater-irrigated soil is wider (the larger the diamond size is the larger the effect size or the difference) (Figure 5).

#### The effect of irrigation duration on irrigated soil

**Figure 6:**
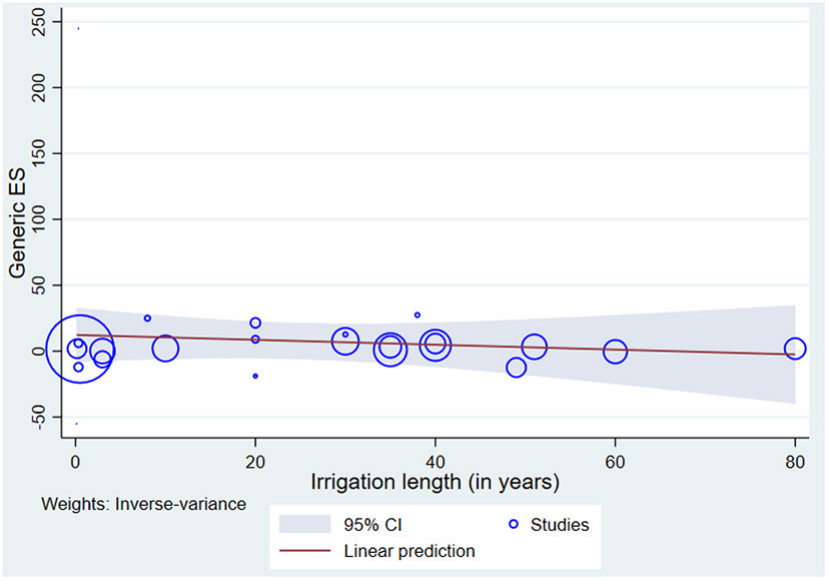
Meta-regression bubble plot of Pb concentration in irrigated soil as a function of the duration of irrigation.

The accumulation of Pb in the soil can be affected by several factors including human activities in the surrounding environment and the duration of irrigation. The effect of duration of irrigation on the accumulation of Pb in irrigated soil was estimated by meta-regression bubble plot of Pb concentration as a function of the duration of irrigation by using random-effect meta-regression model (REML method). The modeling indicates that the association between the concentration of Pb in the irrigated soil and the year of irrigation was not significant (*P* = 0.822) (Figure 6). Based on the regression model equation (SMD = 32.81–0.6245^*^Irrigation Years), as the irrigation duration increases, the effect size gradually decreases. Accordingly, the Pb concentration in clean water irrigated soil (control soil) will be equal to the Pb concentration in wastewater-irrigated soil after 52.54 years of irrigation. However, the model explains only small portions of the changes in the effect size of the studies (R^2^ =7.9%) indicating that Pb concentration in the irrigated soil is moderated by other unexplained factors. This may be probably due to the effect of other operating factors that slows down Pb accumulation in the soil or removal of Pb from the soil or transport of Pb out of the irrigation system. This needs further investigation of the fates of Pb in the irrigated soil.

Moreover, the bubble plot of meta-regression is also used to assess how well the regression model fits the data and to potentially identify influential and outlying studies. The sizes of the markers or the “bubbles,” which are the inverse of the effect size variance, are proportional to the precision of each study. The more precise (larger) the study, the bigger the size of the bubble (63). The predicted regression line and confidence bands are overlaid with the scatter plot. Accordingly, the standardized mean difference (effect size) decreases as the duration of irrigation increases. In our study, the majority of the studies lay within the 95% CI of the prediction line, however, most of the smaller studies are outlying and appear to be less precise, whilst the bigger studies are more precise and within the bands of the confidence interval (Figure 6).

### Concentration of Pb in edible parts of crops

#### Studied crops

**Figure 7:**
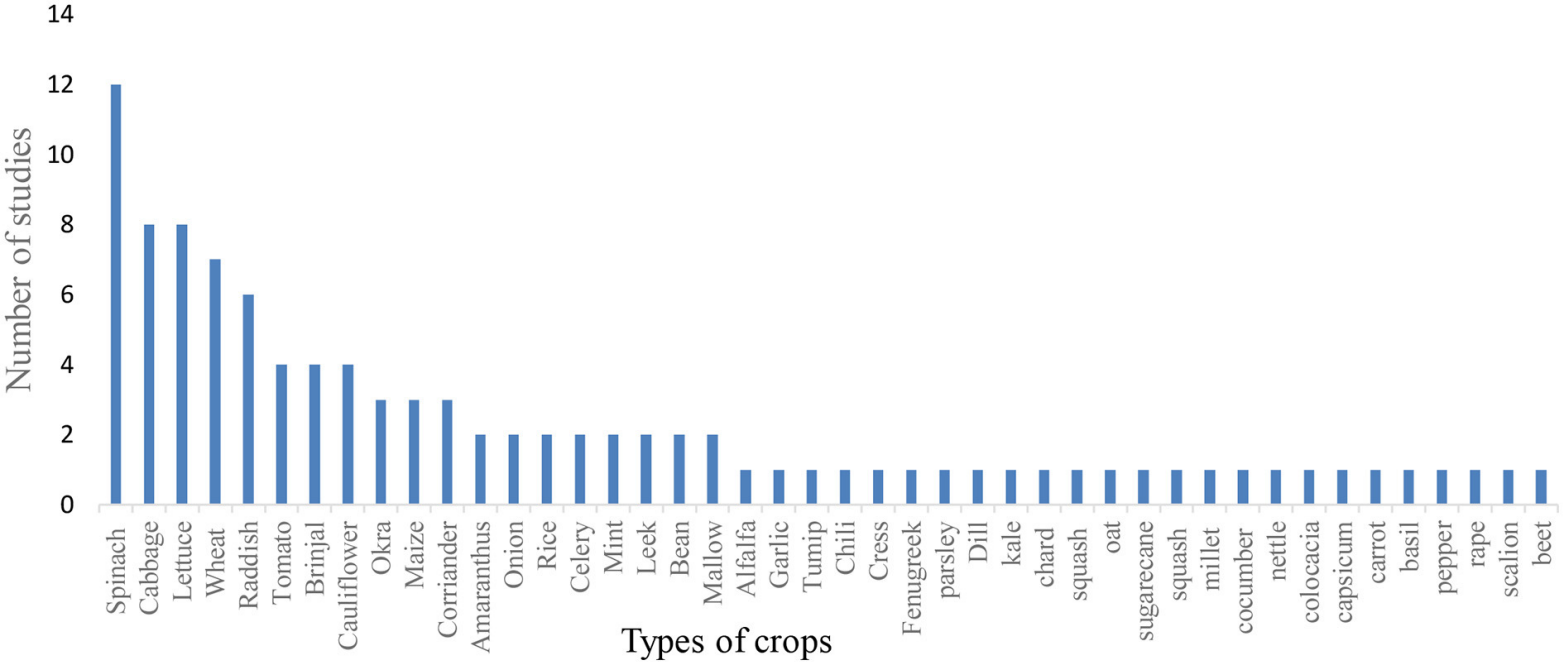
Crops studied by two or more articles.

Figure 7 shows the frequency of crops studied two or more times by the authors of the studies in this meta-analysis. A total of 44 crops were studied by the Twenty-four articles, of which 38 were leafy and non-leafy vegetables, and the rest six were seed crops/grains and others. Twenty-seven of the crops were studied only one time by different authors. Spinach is most frequently selected crop by the researchers (studied in 12 articles), followed by cabbage and lettuce (each studied in 8 articles). The most frequently studied vegetables in this meta-analysis were most popular and among the most important in the world. For instance, spinach is an important leafy vegetable; its leaves and tender shoots are consumed fresh or processed and it is native to central Asia, most probably Iran, the region where most of the studies in a meta-analysis are selected (64). Cabbage is one of the most important and popular vegetables in the world comfortably growing in five continents and in more than 90 countries throughout the world (65).

#### Pb bioaccumulation in crops

The mean Pb concentration in all the crops reported by the studies ranges from 0 mg/kg in maize (3) in a farm field in Iran to a maximum of 2.85 ± 0.92 mg/kg in spinach in Botswana produced from experimental plots by using treated wastewater (41). In untreated wastewater, it ranges from 0.06 ± 0.03 mg/kg in celery in Iran (44) to a maximum of 370.43 ± 42.27 mg/kg in the edible leaves of mallow in Egypt (52) followed by cauliflower (86.69 + 6.69) and spinach (68.17 + 4.96) in India (53). The Pb concentration in all of the crops grown by using raw wastewater is much higher than the Pb concentration in crops grown by using treated wastewater; whereas all the studies carried out using treated wastewater, the Pb concentration is below the limit set by FAO and WHO (0.3 mg/kg). Though the treatment technologies are not mentioned in any of the studies, it is clear that during treatment process the Pb in the wastewater may be removed efficiently so that the Pb availability for plant absorption is limited.

**Figure 8:**
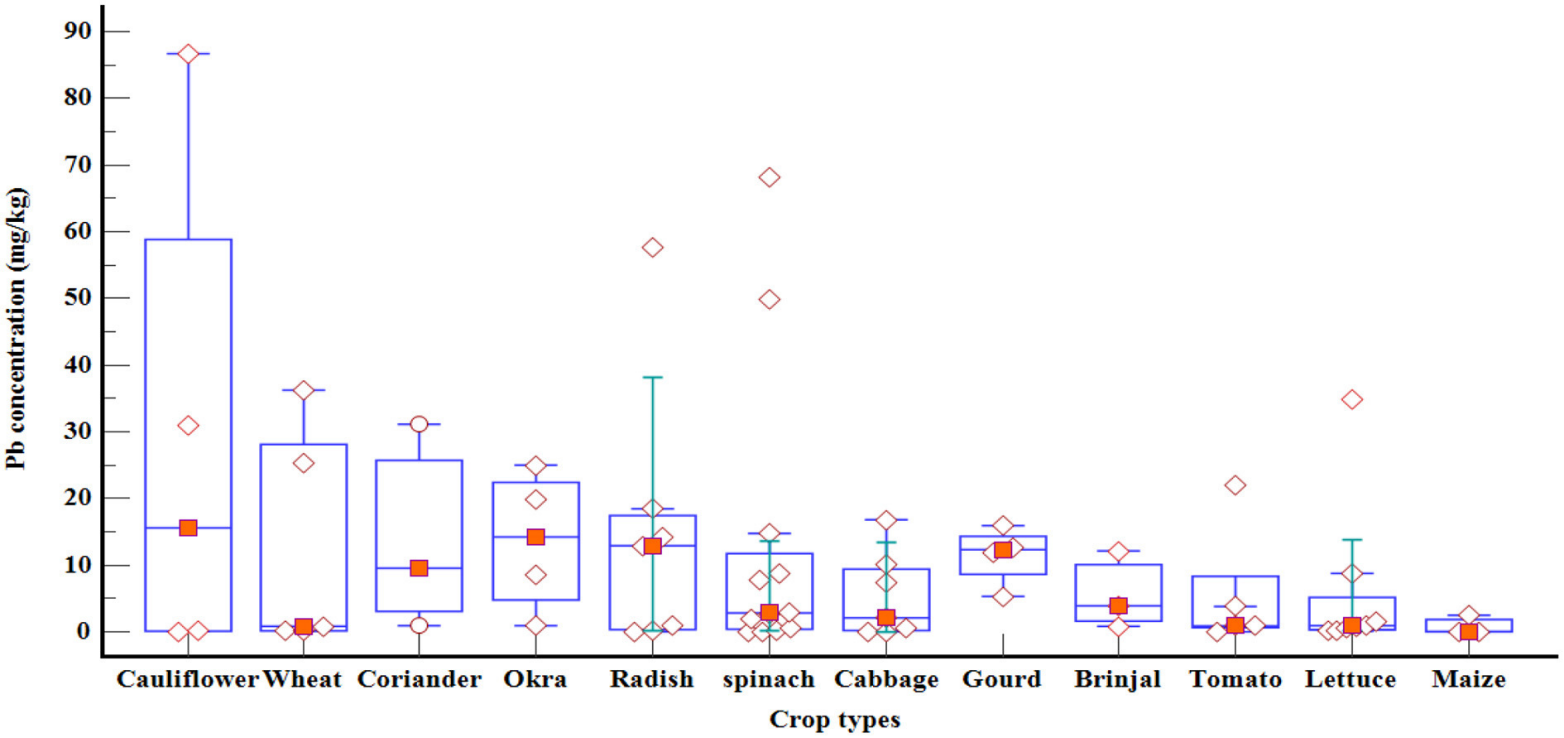
Box and whisker plot of Pb concentration in twelve crops (studied three to twelve times by different authors). The diamonds represents individual studies.

The box and whisker plot shows the dispersion of the data for the Twelve most frequently studied crops, which are studied 3 to 12 times, by using the five-number summary [minimum, first quartile (Q1), median, third quartile (Q3), and maximum] (Figure 8). The graph clearly shows the wide variabilities of Pb concentration between studies. Among these crops, the maximum concentration (86.69 ± 6.69 mg/kg) is recorded in cauliflower in India followed by spinach (68.17 mg/kg) in an experimental field in Botswana. As spinach is the most studied crop, wide and more variabilities in Pb concentration is observed. Among most studied crops, the order of the crops by the mean and maximum values is cauliflower ->wheat->coriander->okra->radish->spinach->brinjal->cabbage->gourd- lettuce->tomato->maize.

Accumulation of toxic heavy metals in edible food crops is a potential threat to human and animal health. Hence, studies on soil and food crop relationships in terms of heavy metals accumulation are expedient. Crops have different potential to absorb and accumulate heavy metals in their edible parts.

### Health risk assessment

#### Pollution (Pb) load index (PLI)

Accumulation of heavy metals in irrigated soils due to wastewater irrigation results in soil contamination and also leads to elevated uptake by crops, and thus affects food quality and safety (66). Table 3 shows PLI, and (SAF reported by the authors.

**Table 3:** Lead load index and soil lead accumulation factor.

	**Author**	**Location**	**PLI**	**SAF**
1	Ahmed and Slima (52)	Egypt	75.6	1.4
2	Asgari and Cornelis (3)	Iran	1.074	1013.5
3	Chaoua et al. (67)	Morocco	1.55	40.39
4	Chopra and Pathak (53)	India	3.99	223.76
5	Chung et al. (37)	N. korea	1.03	2.27
6	Gupta et al. (38)	India	12.54	30.62
7	Khan et al. (23)	Pakistan	2.88	7.34
8	Khan et al. (4)	China	18.36	49400
9	Kharazi et al. (39)	Iran	3.49	1.4
10	Kim et al. (40)	S. Korea	0.76	199.5
11	Letshwenyo et al. (41)	Botswana	89.52	7050
12	Mahfooz et al. (42)	Pakistan	1.184	9.07
13	Mustapha and Adeboye (43)	Nigeria	1.6	111.11
14	Qishlaqi et al. (44)	Pakistan	6.99	13385
15	Rezapour et al. (45)	Iran	1.63	3.143
16	Sharma et al. (54)	India	0.34	64.73
17	Sayo et al. (46)	Kenya	20.68	0.384
18	Tariq (47)	Iraq	2.1	278.89
19	Tiwari et al. (6)	India	22.62	2.7
20	Ullah et al. (49)	Pakistan	2.18	46.07
21	Ullah and Khan (48)	Pakistan	2.09	35.61
22	Waheed et al. (55)	Pakistan	1.91	0.5
23	Wang et al. (50)	China	4.17	81.56
24	Woldetsadik et al. (51)	Ethiopia	0.002	2.24

The majority of the studies, although they have the necessary data, they did not calculate the soil Pb load index. For this study, the PLI is calculated for each study to show Pb accumulation potential in the farmland because of the use of wastewater for irrigation. The PLI was ranged from a minimum of 0.002 in leafy vegetable irrigation soil (51) to a maximum of 89.52 in spinach irrigation soil (41) and 75.6 in Jew mallow (52). In all of the studies, the PLI is higher than one, indicating that there is a buildup of Pb concentration in wastewater-irrigated soil. However, PLI is weakly negatively correlated with durations of irrigation (*r* = – 0.358, *P* = 0.086) implying that the Pb accumulation in wastewater irrigated soil compared to the reference soil decreases with how long the soil is irrigated. The median value is 2.09 indicating that in fifty percent of the studies the Pb load index is well below the mean value. Apart from the two-outlier Pb loads in the two experimental plots in Botswana and Egypt, four studies are outliers, which is beyond the maximum value.

#### Soil accumulation factor

Soil accumulation factor is the measure of the capacity of the soil to adsorb/take up pollutants from the wastewater. SAF ranged from a minimum of 0.384 in an experimental field plot irrigated with wastewater in Kenya (46) to a maximum of 49,400 in a study carried out in China (4). Most of the studies, particularly studies on vegetables, illustrate very high levels of SAF indicating that Pb concentration build-up is a common phenomenon in wastewater-irrigated soil (Table 3).

Although SAF is expected to associate with durations of irrigation, the Pearson correlation coefficient shows a very weak association of SAF with how long the farm is irrigated (*r* = 0.085, *P* = 0.693). This may be due to the rapid saturation of the adsorption site on the soil by the rapid accumulation of Pb, which gradually decreases and stops accepting more Pb over time. Once the adsorption sites on the soil surface are rapidly occupied, Pb adsorption may slow down to equilibrium conditions. The wide variation in the values of SAF could be due to differences in factors affecting the accumulation of lead in each soil type, lead concentration in the wastewater, differential absorption by the crops and other environmental factors. Thus, the extremely large SAF values in some studies such as Asgari and Cornellis (1013.5), Letshwenyo et al. (7050), Qishlaqi et al. (13,385) and Khan et al. (49,400) might be due to the presence of facilitating conditions for lead adsorption by the soil.

#### Plant concentration factor

**Figure 9:**
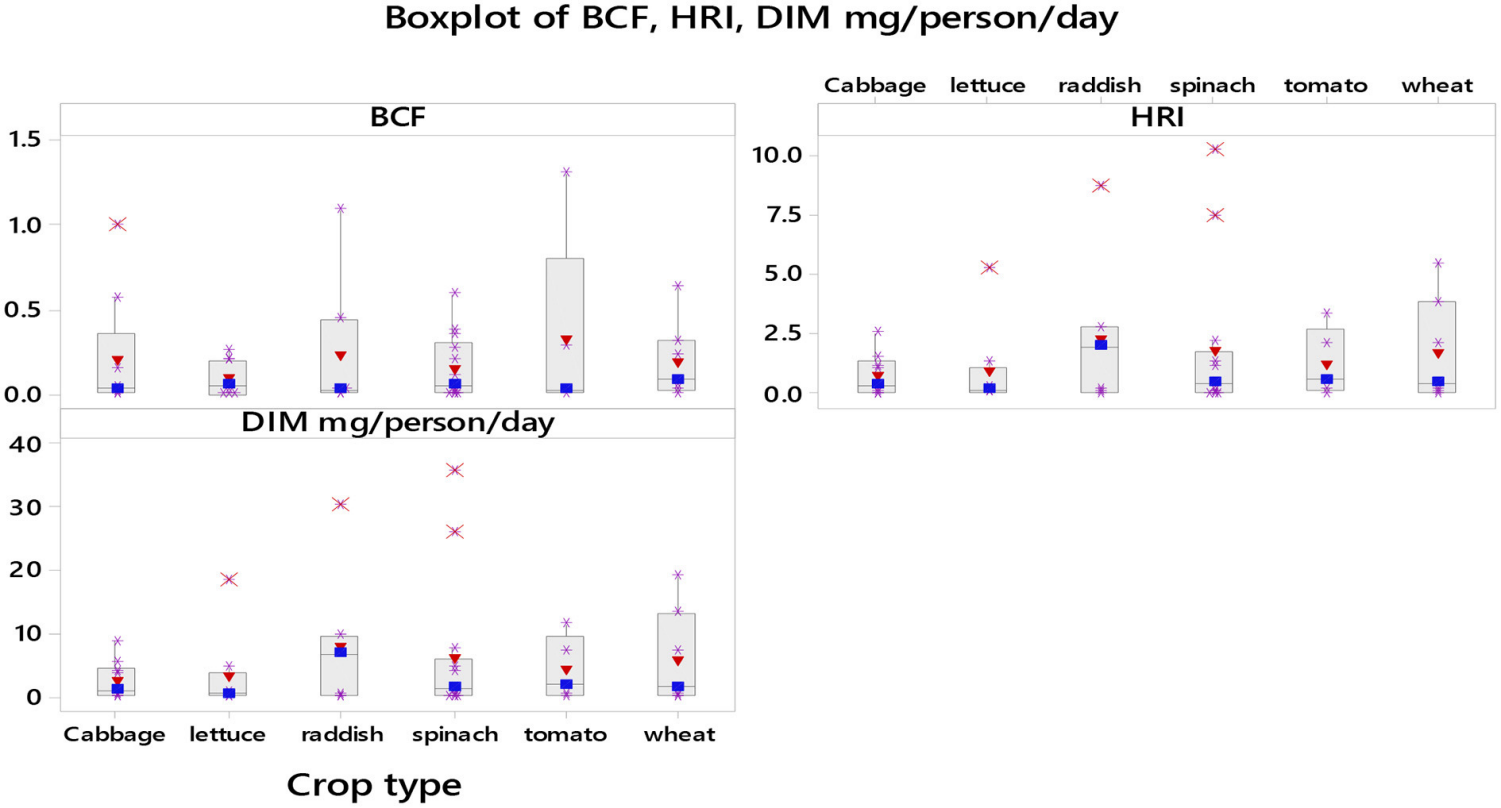
Plant concentration factor (PCF/BCF) (The maximum PCF value (5) for one study is not included in this graph) and daily intake of lead (DIM) and its health risk index (HRI).

Figure 9 shows the bioconcentration factor, daily intake of Pb and HRI of Pb through the consumption of six most used wastewater-produced vegetables. The bioconcentration factor (PCF or BCF) or transfer factor (TF) from soil to crops is one of the roots of human exposure to metals through the food chain. To evaluate the HRI associated with wastewater-irrigated soils, it is essential to assess the TF (68). The bioaccumulation of metals in plants from soils can be predicted using a transfer factor (TF) or bioconcentration factor (PCF) (69).

The PCF ranges from 0 in six crops (maize, celery, squash, tomato, cucumber, and Chinese cabbage) to 5 in Jew mallow (52) followed by 1.49 in okra and 1.44 in pallak (70). Accumulation of Pb in different parts of edible plants is very common. Among the study plants, wheat, maize, onion, and mint accumulated equal or higher than the amount accumulated in the soil where they grew (23, 43, 71). The order of vegetables based on their PCF is jew mallow > okra > tomato > amaranths> eggplant > cabbage > radish. This implies that consuming Jew mallow, okra or tomato can have more risk of Pb exposure than the other crop types. Typically, the soil-to-plant transfer factor is one of the key components of human exposure to metals through the food chain. To investigate the human risk associated with wastewater-irrigated soils, it is essential to assess the PCF (68). The high transfer values of Pb from soil to plants for some vegetables indicate a strong accumulation potential of Pb by the food crops, particularly by leafy vegetables.

Studies show that the transfer coefficient may be varied considerably between plant, soil, and metal types under investigation (72). The differences in accumulating capacity may be related to the differential Pb-binding capacity of the vegetables (73), interactions between physicochemical parameters, and the plant species grown in these soils (74). Moreover, the absorption and accumulation of heavy metals in plant tissues depend upon many complex factors including temperature, moisture, organic matter, pH, and nutrient availability, for instance, the presence of organic matter has been reported to increase the uptake of Pb in the wheat plant (75). A study also reported that the variations in transfer factor of metals in different vegetables is related to each vegetable's absorption capability, soil nutrient management, and soil properties (76). Therefore, by choosing suitable crops, the risk of human exposure to metal contamination can be considerably reduced (77).

To assess the human health risk of any heavy metal (Pb), it is essential to estimate the level of exposure by quantifying the routes of exposure of humans to various Pb levels. Though there are various possible exposure pathways, the consumption of Pb-contaminated food is the most important.

#### Health risk index

The HRI represents the harmful effect of Pb to people consuming vegetables contaminated with heavy metals. If the value of HRI is less than one, people will be safe to eat those kinds of vegetables (32). As mentioned above, food crops were contaminated with Pb metals and the consumption of such kinds of stuff can cause human health risks. Figure 9 shows the data distribution of daily intake and HRI of Pb. Daily intake of lead (DIM) ranges from 0 in maize in Iran to 194.5 mg/person/day in Jew mallow in Egypt.

HRI of the studies ranges from 0 in maize (3) to 55.56 in Jew mallow (52) followed by 13 in cauliflower (53). The HRI in all the studies included in this meta-analysis from India is beyond one indicating that no studied vegetables are safe for human consumption. Moreover, the HRI calculated in Morocco (2–7.93), Iraq (4.42–4.6 except cress) and Egypt (55.57) also showed that the studied vegetables are not safe for human consumption.

## Conclusion

Most studies are carried out only in four nations and used raw wastewater as irrigation water source. The Pb concentration in all of the contaminated irrigation water is much higher than the limits of irrigation water standard set by WHO. Similar to the Pb concentration in the irrigation wastewater, the Pb level in irrigated soil varies considerably and much higher than the limits. However, few smaller studies did not show statistically significant difference between contaminated and non-contaminated soil. The heterogeneity test statistics indicates that the studies are considerably heterogeneous. The subtotal effect sizes of each subgroup show that there is significant between-study differences in both raw wastewater and treated wastewater groups. The association between the concentration of Pb in the irrigated soil and the year of irrigation was not significant, but as irrigation duration increases effect size gradually decreases.

The most common vegetables including spinach, cabbage, lettuce and okra contain are frequently studied crops and contain high levels of Pb in their edible parts of the crops. The Pb concentration in all of the crops grown by using raw wastewater is much higher than the Pb concentration in crops grown by using treated wastewater and beyond the acceptable limit. Among most studied crops, the order of the crops by the mean and maximum values is cauliflower > wheat > coriander > okra > radish > spinach > brinjal > cabbage > lettuce > tomato > maize. Crops have different potential to absorb and accumulate heavy metals in their edible parts high enough to be human health risk. The PLI and SAF analysis show that there is continuous buildup of Pb concentration in irrigated soil high enough to be absorbed by vegetables. According to the DIM and HRI values, all those studies from India, Iraq, Egypt and Morocco show that eating those studied vegetables are not safe.

## Data availability statement

The raw data supporting the conclusions of this article will be made available by the authors, without undue reservation.

## Author contributions

All authors listed have made a substantial, direct, and intellectual contribution to the work and approved it for publication.

## Conflict of interest

The authors declare that the research was conducted in the absence of any commercial or financial relationships that could be construed as a potential conflict of interest.

## Publisher's note

All claims expressed in this article are solely those of the authors and do not necessarily represent those of their affiliated organizations, or those of the publisher, the editors and the reviewers. Any product that may be evaluated in this article, or claim that may be made by its manufacturer, is not guaranteed or endorsed by the publisher.
